# Type 1 Diabetic Subjects with Diabetic Retinopathy Show an Unfavorable Pattern of Fat Intake

**DOI:** 10.3390/nu10091184

**Published:** 2018-08-29

**Authors:** Minerva Granado-Casas, Anna Ramírez-Morros, Mariona Martín, Jordi Real, Núria Alonso, Xavier Valldeperas, Alicia Traveset, Esther Rubinat, Nuria Alcubierre, Marta Hernández, Manel Puig-Domingo, Albert Lecube, Esmeralda Castelblanco, Didac Mauricio

**Affiliations:** 1 Department of Endocrinology and Nutrition, Health Sciences Research Institute & University Hospital Germans Trias i Pujol, 08916 Badalona, Spain; mgranado@igtp.cat (M.G.-C.); aramirez@igtp.cat (A.R.-M.); nalonso32416@yahoo.es (N.A.); mpuigd@igtp.cat (M.P.-D.); 2 Lleida Institute for Biomedical Research Dr. Pifarré Foundation, IRBLleida, University of Lleida, 25198 Lleida, Spain; atravesetm@gmail.com (A.T.); cubito@lleida.org (N.A.); martahernandezg@gmail.com (M.H.); alecube@gmail.com (A.L.); 3 Department of Endocrinology & Nutrition, University Hospital Germans Trias i Pujol, 08916 Badalona, Spain; marionamarting@gmail.com; 4 Epidemiology and Public Health, International University of Catalonia, 08017 Barcelona, Spain; jordireal@gmail.com; 5 Unit Support of Research, Institut d’Investigació en Atenció Primària Jordi Gol (IDIAP Jordi Gol), 08007 Barcelona, Spain; 6 Center for Biomedical Research on Diabetes and Associated Metabolic Diseases (CIBERDEM), Instituto de Salud Carlos III, 08907 Barcelona, Spain; rubinatesther@gmail.com; 7 Department of Ophthalmology, University Hospital Germans Trias i Pujol, 08916 Badalona, Spain; xvalldeperas@gmail.com; 8 Department of Ophthalmology, University Hospital Arnau de Vilanova, 25198 Lleida, Spain; 9 Department of Nursing and Physiotherapy, University of Lleida, 25198 Lleida, Spain; 10 Department of Endocrinology & Nutrition, University Hospital Arnau de Vilanova, 25198 Lleida, Spain; 11 Department of Endocrinology & Nutrition, Hospital de la Santa Creu i Sant Pau, Autonomous University of Barcelona, 08041 Barcelona, Spain

**Keywords:** type 1 diabetes, diabetic retinopathy, dietary pattern, oleic acid, fatty acids, carbohydrates

## Abstract

Medical nutrition therapy is an important part of the management of type 1 diabetes mellitus (T1DM). Proper adherence to a healthy diet may have a favorable impact on diabetes management and its diabetic complications. Our aim was to assess differences in food and nutrient intake of type 1 diabetic patients with and without diabetic retinopathy (DR). This was a two-center, cross-sectional study in patients with T1DM, with and without DR. Subjects were recruited from the outpatient clinic of the two participating centers. A validated food frequency questionnaire was administered. A total of 103 T1DM patients with DR and 140 T1DM patient without DR were recruited. Subjects with DR showed a lower intake of total fat (*p* = 0.036) than that of their non-DR counterparts. DR was associated with increasing age (*p* = 0.004), hypertension (*p* < 0.001), and diabetes duration (*p* < 0.001), however there was a negative association with high educational level (*p* = 0.018). The multivariate-adjusted analysis showed that the intake of complex carbohydrates was positively related to the presence of DR (*p* = 0.031). In contrast, the intakes of total fat (*p* = 0.009), monounsaturated fatty acids (MUFAs) (*p* = 0.012), oleic acid (*p* = 0.012), and vitamin E (*p* = 0.006) were associated with the absence of DR. As conclusions, the intake of total MUFAs, oleic acid, and vitamin E is associated with a lower frequency of DR in patients with T1DM. These results suggest a potential protective effect of these lipid components for DR.

## 1. Introduction

Diabetic retinopathy (DR) is an important cause of visual impairment in patients with type 1 diabetes mellitus (T1DM) [1]. According to a previous study in a cohort of patients with T1DM, the 10-year incidence of DR was 35.9% [2]. The American Diabetes Association (ADA) and European Association for the Study of Diabetes (EASD) establish that medical nutrition therapy (MNT) plays an important role in preventing complications related to the management and metabolic control of the disease [3]. These nutritional recommendations are based on the Mediterranean Diet, which has benefits to prevent cardiovascular diseases in the diabetic population. Additionally, a positive relationship between severity of DR and modifiable risk factors such as hypertension, dyslipidemia, and smoking has been described in T1DM [1].

Currently, a controversy exists with the few published studies, regarding the relationship between the dietary intake and the presence of DR in patients with diabetes, describing controversial results between them [4,5]. The relationship between dietary intake and DR development in patients with T1DM has only been described in few studies [6,7,8,9,10,11,12]; specifically, there are only seven studies that have assessed the relationship between dietary intake and the prevalence of DR in patients with T1DM [6,7,8,9,10,11,12]. Of these, four studies related alcohol consumption and/or salt intake with the frequency of DR in a cross-sectional study design [9,10,11,12]. Results of the Diabetes Control and Complications Trial (DCCT) study cohort pointed to higher risk of DR progression in those subjects with higher intake of fatty acids, and a lower-risk association among those with higher dietary fiber intake [6]; however, another study did not find any differences [7]. A study with a cross-sectional design and a sample of 379 patients with diabetes mellitus (of whom only 52 had T1DM) observed a protective effect of MUFA and polyunsaturated fatty acid (PUFA) intake for DR in well-controlled patients [8]. The EURODIAB Study only examined the relationship between alcohol consumption and salt intake with the risk of DR in T1DM patients [9,10]. They found a lower risk of DR with a moderate alcohol consumption [9]; nevertheless, no association was observed with an increased salt intake with DR in this diabetic population [10]. On the other hand, the FinnDiane Study [11] and Moss et al. [12] found an inverse association between alcohol consumption and the presence of DR in these patients. According to two recently published systematic reviews about the relationship between dietary intake and DR, no study has assessed the relationship between the whole food and nutrient intake and DR in patients with T1DM [4,5]. 

In our region, apart from receiving specific education on carbohydrate counting learning, medical nutritional therapy for diabetic subjects includes recommendations to adopt a Mediterranean Diet pattern [13,14]. To our knowledge, there are no studies designed to compare the relationship between the food and nutrient intake in T1DM patients with the presence or absence of DR. In addition, our group described a positive association between the higher intake of MUFAs and oleic acid and a lower frequency of DR in patients with T2DM [15]. For this reason, we hypothesized that patients with T1DM and DR have a lower adherence to the nutritional recommendations than that of their counterparts without DR. Therefore, the aim of the current study was to assess differences in the food and nutrient intake of patients with T1DM, with and without DR.

## 2. Materials and Methods

This was a cross-sectional, two-center study. The participants were regularly cared for at their reference hospital (University Hospital Arnau de Vilanova and University Hospital Germans Trias i Pujol, both in Northeastern Spain). The study participants were recruited from the health care districts of Lleida (Center 1) and Badalona (Center 2). Lleida is a rural and semi-urban region, while Badalona is an urban location in the metropolitan area of Barcelona. This was a post hoc analysis from a previously published study [14]; from a sample of 259 patients with T1DM, 16 patients were excluded because they did not have a full assessment of DR status. A final sample of 103 T1DM patients with DR and 140 T1DM without DR was included. More details of the original study are available in [14]. The recruitment was performed between January 2013 and May 2015. As inclusion criteria, we used the following: T1DM with a duration of at least one year and age over 18 years. The exclusion criteria were as follows: patients who were healthcare professionals; patients who showed physical or cognitive deterioration (e.g., dementia, mental diseases); history of clinical cardiovascular disease or diabetic foot disease, pregnancy, renal insufficiency (estimated glomerular filtration rate <60 mL/min); and patients with a condition that required additional MNT measures (i.e., macroalbuminuria defined as urine albumin/creatinine ratio >299 mg/g).

The ethics committees of the two participating centers approved the study, and written informed consent forms were obtained from all participants (PI-13-095 and PI-15-147). 

### 2.1. Clinical Variables

Clinical variables were collected through detailed anamnesis, physical exam, and careful review of the clinical records. Hypertension and dyslipidemia were defined by the use of any specific medication for the given conditions. Blood and urine chemistry variables were determined by standardized methods. Physical activity was assessed by the validated method of Bernstein et al. [16] and Cabrera de León et al. [17]. A subject was classified as having regular physical activity if she/he performed more than 25 min/day of any type of physical activity that requires 4 METS (the metabolic equivalent) minimum. Participants were classified as sedentary if they did less than 25 min/day of any physical activity.

### 2.2. Dietary and Nutritional Assessment

A food frequency questionnaire (FFQ) was administered to all participants of the study during personal interviews by specialized and trained researchers. This was a semi-quantitative questionnaire, validated and adapted for the Spanish population, based on the Nurses’ Health Study (available at: http://epinut.edu.umh.es/wp-content/uploads/sites/1365/2011/07/CFA101.pdf) [18,19]. The questionnaire contained 101 items, in which it asked for their usual consumption since the previous year’s visit. Serving sizes were detailed for each food item in the FFQ. The questionnaire had nine possible answers, ranging from “never or less than once per month” to “six or more per day”. Nutrient values were primarily obtained from the food composition tables of the US Department of Agriculture and other published sources for Spanish and English foods and portion sizes [20,21,22]. We used the Spanish food composition tables to avoid an overestimation of nutrient intake from the fortified dairy products of the US.

### 2.3. Assessment of Diabetic Retinopathy

Retinopathy was assessed and classified by an ophthalmologist according to the international clinical classification system [23]. The ophthalmologist performed a complete eye evaluation and defined DR in five stages: (1) no apparent retinopathy, (2) mild non-proliferative retinopathy, (3) moderate non-proliferative retinopathy, (4) severe non-proliferative retinopathy, and (5) proliferative diabetic retinopathy.

### 2.4. Statistical Analysis

Descriptive comparison between groups of all variables was performed to evaluate the differences. Statistical significance was assessed by the chi-square test, where the frequencies of comorbidities and demographic variables were compared among groups. The mean differences between groups were compared by Student’s *t*-test. Methods of Benjamini, Hochberg, and Yekutieli were performed to control the false discovery rate and expected proportion of false discoveries among the rejected hypotheses. Conditional logistic regression models were developed to analyze the relationship between the presence of diabetic retinopathy (DR), its clinical characteristics, and daily nutrient intake. Crude and adjusted odds ratios (ORs) were estimated by univariable and multivariable conditional logistic regression models, respectively. Adjusted odds ratios were fitted by age, sex, educational level, smoking, center, physical activity, body mass index, dyslipidemia, hypertension, and diabetes duration. In all models, the goodness-of-fit assumption was tested by the Hosmer–Lemeshow test. Estimates of odds ratios were reported with corresponding 95% confidence intervals and statistical significance was established as a *p*-value < 0.05. Data management and analyses were performed with the free software environment R version 3.3.2 and SPSS software (version 20, SPSS, Chicago, IL, USA).

## 3. Results

Clinical characteristics of the participants are shown in Table 1. Patients with DR demonstrated the following: were older (*p* = 0.010), had higher systolic blood pressure (*p* = 0.005) and frequency of hypertension (*p* < 0.001), had longer duration of diabetes (*p* < 0.001) and showed worse glycemic control (*p* < 0.001) and lipid profile, and had lower HDL (*p* = 0.045) and higher triglycerides (*p* = 0.045).

Concerning daily nutrient intake, patients with DR had a lower fat intake than those without DR did (*p* = 0.036) (Table 2). Although not significantly different, the intake of MUFAs, oleic acid, and vitamin E was lower in patients with DR than in those without (*p* = 0.050). In terms of daily food intake, no statistical differences were found between patients with and without DR (Table S1). However, a tendency to eat more bread (*p* = 0.072) and less vegetable fat (*p* = 0.072) was observed in patients with DR.

In the bivariate analysis, including the clinical characteristics (Table 3), higher educational level was associated with the absence of DR (OR: 0.47; 95% CI: 0.25–0.88; *p* = 0.018). In contrast, DR was associated with increasing age (OR: 1.04; 95% CI: 1.01–1.06; *p* = 0.004), hypertension (OR: 3.77; 95% CI: 2.05–7.13; *p* < 0.001), diabetes duration (OR: 1.10; 95% CI: 1.07–1.14; *p* < 0.001), and glycemic control (OR: 1.97, 95% CI: 1.44–2.69; *p* = 0.001).

In the crude multivariate analysis for nutrient intake, a high intake of complex carbohydrates (*p* = 0.031) was associated with the presence of DR (Table 4). On the other hand, the absence of retinopathy was associated with a higher intake of total fat (*p* = 0.009), MUFAs (*p* = 0.012), oleic acid (*p* = 0.012), and vitamin E (*p* = 0.006). In the multivariate adjusted model, only the intake of complex carbohydrates (*p* = 0.031) remained associated with the presence of DR, whereas the intake of total fat (*p* = 0.009), MUFAs (*p* = 0.012), oleic acid (*p* = 0.012), and vitamin E (*p* = 0.006) were still associated with the absence of DR.

## 4. Discussion

Our results suggest that the intake of total fat, MUFAs, oleic acid, and vitamin E is associated with the absence of DR in subjects with T1DM. In contrast, the intake of complex carbohydrates is related to the presence of DR. To our knowledge, this is the first study that has so far assessed the overall food and nutrient intake in patients with T1DM with DR.

In terms of total fat and MUFA intake, the present results are discordant with the results of a sub-study of the DCCT trial, which found an association between the increased intake of fat, SFAs and MUFAs and the presence of DR [6]; however, these researchers did not adjust for potential confounders [5], i.e., age, sex, smoking, diabetes duration, glycated hemoglobin (HbA1c), body mass index (BMI), and the presence of hypertension or dyslipidemia. In addition, Roy et al. [7] did not find any association between the intake of fatty acids and the risk of 6-year progression of DR in a large cohort of T1DM subjects who consumed a high amount of SFAs, PUFAs, and dietary cholesterol. This result was in line with the results of a cross-sectional study, including well-controlled patients with T1DM and T2DM [8]. On the other hand, a protective association with higher intake of MUFAs and oleic acid was observed in a previous study in patients with T2DM performed by our group, a finding that was similar with the current results [15]. The PREDIMED trial showed that a Mediterranean diet supplemented with extra virgin olive oil (a MUFA-rich diet) and a PUFA-rich diet were related with a lower incidence of DR at follow-up in patients with T2DM [24,25]. Moreover, a cross-sectional study observed that the intake of PUFAs was a protective factor of DR in well-controlled diabetic patients [8]. However, the DCCT study found that an intake of PUFAs was related with a high risk of DR [6]; no such association was shown in another prospective study in patients with T1DM [7], a finding that is similar to our results. These two prospective studies are contrary with our results. Two meta-analyses of randomized clinical trials showed that a high-MUFA diet improves glycated hemoglobin and fasting blood glucose in patients with diabetes when compared with that of a low-MUFA diet or high-carbohydrate diet [26,27]; furthermore, MUFAs have potential benefits in the lipid profile, i.e., increasing high-density lipoprotein (HDL) serum levels in diabetic patients [27]. This result has great importance because a MUFA-rich diet may have the potential to prevent diabetic complications long-term. A recent review on the role of omega-3 in the development of DR described that α-linolenic acid improves the progression of DR by means of various antioxidant and anti-inflammatory mechanisms in animal and human studies [28].

Interestingly, our results showed that a higher intake of carbohydrates was positively related with the presence of DR in patients with T1DM. This finding is in contrast with those of the DCCT study, which found a lower presence of DR with a high intake of carbohydrates and dietary fiber in adolescents and young adult patients with T1DM [6]. However, the other few studies that have assessed the possible association between dietary carbohydrates and DR did not find such association [7,8,15,29]. In line with our results, the EURODIAB study described that a rich-carbohydrate diet derived from complex carbohydrates (not dietary fiber) produces negative effects in glycemic control and consequently a high risk of diabetic complications [30].

Our findings indicate that a higher intake of vitamin E is associated with a lower presence of DR; this result is due to a high intake of vegetable fats. Although a risk association between the intake of vitamin E and DR was found in a cross-sectional study including a large sample of patients with T2DM [31], some prospective and other cross-sectional studies did not confirm this association [8,32,33]. This finding should be further investigated due to controversy in the scientific studies.

In terms of alcohol consumption, we did not find any difference between the two groups; however, two cross-sectional studies performed in patients with T1DM found a positive relationship between alcohol consumption and the presence of DR [9,11]; and another cross-sectional study showed that an increased alcohol consumption was related to a lower prevalence of DR in younger-onset diabetes [12]. Therefore, we must gain more insight into this potential dietary contributing factor.

Moreover, the current study showed that a higher educational level was related with a lower presence of DR; this result is similar to the those of the EPIC study, which associated a high educational level with better dietary habits in Mediterranean countries [34]. This result points to the possible link between a healthier dietary intake and a lower prevalence of DR in diabetic subjects.

This study has some limitations. First, the association of causality between the intake of nutrients and the presence of DR cannot be established because of the cross-sectional study design. For this reason, no specific nutritional recommendations to the patients can be derived from our results. Furthermore, the changes in dietary habits over time cannot be determined with this study. Another limitation of our study is that we did not assess the specific knowledge of each study participant on the dietary management of diabetes and its relationship with the individual food intake. Therefore, we could not evaluate whether patients with DR had a less favorable dietary fat intake as a result of poorer adherence and/or lower knowledge of dietary measures. Moreover, this is a sub-study of a previous study designed to assess differences in the Mediterranean diet between patients with T1DM and the non-diabetic population [14]. However, this study has several strengths. This is the first study that assessed the relationship between the overall dietary intake and the prevalence of DR in patients diagnosed with T1DM. Additionally, we included a large number of participants in this multicenter study with a very well-defined sample that allowed us to establish the variability of different populations and regions. The use of the FFQ allowed us to define the proper estimation of nutrients and food intake of patients. Patients with T1DM receive nutritional education for the management of the disease over the time. In our region, the MNT is based on the nutritional recommendations of the Mediterranean diet. Therefore, we can assume that the patients had maintained the dietary habits over a long period of time; among other factors, a higher adherence to a healthy dietary pattern may help to prevent diabetes-related complications. In addition, this FFQ has been shown to be representative of the previous five-year period of the subject’s food intake [35]. However, the cross-sectional design does not allow us to draw a final conclusion. Finally, we can consider that the present results could be potentially important for future research because there has been no other study that specifically assesses food and nutrient intake with the prevalence of DR in patients with T1DM.

## 5. Conclusions

In conclusion, a lower intake of total fat, MUFAs, oleic acid, and vitamin E was shown to be associated with the presence of DR. However, a lower intake of complex carbohydrates was associated with DR absence. Further research is necessary to establish associations and causality between dietary patterns and the presence of DR in this population.

## Figures and Tables

**Table 1:** Clinical characteristics of participants according to diabetic retinopathy status.

Characteristics	DR (*n* = 103)	No DR (*n* = 140)	*p* ^1^
Age (years)	46.2 ± 10.8	42.1 ± 10.3	0.010
Sex (male)	48 (46.6)	62 (44.3)	0.867
Educational level			0.107
Not even primary	4 (4.1)	8 (5.9)	
Completed primary	36 (36.0)	34 (25.2)	
Secondary high school	39 (40.2)	49 (36.3)	
Graduate or higher	18 (18.6)	44 (32.6)	
Smoking			0.602
No	48 (46.6)	73 (52.1)	
Yes	32 (31.1)	34 (24.3)	
Former smoker	23 (22.3)	33 (23.6)	
Regular physical activity	77 (74.8)	98 (70.0)	0.602
Waist circumference (cm)	90.7 ± 13.4	87.0 ± 12.1	0.075
Systolic blood pressure (mmHg)	131.0 ± 18.4	123.2 ± 16.1	0.005
Diastolic blood pressure (mmHg)	73.8 ± 9.3	74.2 ± 9.6	0.846
BMI (kg/m^2^)	26.2 ± 4.3	25.3 ± 4.0	0.111
Hypertension	40 (38.8)	20 (14.3)	<0.001
Dyslipidemia	49 (47.6)	49 (35.0)	0.107
Diabetes duration (years)	26.5 ± 9.9	17.9 ± 9.1	<0.001
HbA1c (%)	7.9 ± 1.1	7.4 ± 0.8	<0.001
HbA1c (mmol/mol)	63.0 ± 11.9	56.9 ± 8.3	<0.001
Total cholesterol (mg/dL)	179.0 ± 30.8	182.3 ± 27.1	0.602
HDL cholesterol (mg/dL)	61.9 ± 16.8	66.8 ± 14.9	0.045
LDL cholesterol (mg/dL)	102.7 ± 25.5	102.2 ± 22.7	0.993
TG (mg/dL)	81.4 ± 49.9	68.7 ± 26.7	0.045

Data are means ± SD or n (%). ^1^ *p* was calculated by method of Benjamini and Hochberg. DR, diabetic retinopathy; HbA1c, glycated hemoglobin; BMI, body mass index; HDL cholesterol, high density lipoprotein cholesterol; LDL cholesterol, low density lipoprotein cholesterol; TG, triglycerides.

**Table 2:** Daily nutrient intake of participants according to diabetic retinopathy status.

Daily Nutrient Intake ^1^	DR (*n* = 103)	No DR (*n* = 140)	*p* ^2^
Energy intake (kcal)	2047.0 ± 556.0	2077.7 ± 482.3	0.796
Carbohydrate (g)	198.0 ± 36.3	186.6 ± 31.2	0.075
Complex carbohydrate (g)	91.6 ± 18.6	85.8 ± 18.1	0.096
Sugar (g)	82.0 ± 24.3	78.9 ± 23.1	0.604
Fiber (g)	23.4 ± 5.8	22.4 ± 6.2	0.545
Soluble fiber (g)	3.6 ± 1.3	3.5 ± 1.2	0.604
Insoluble fiber (g)	13.6 ± 4.4	12.9 ± 4.1	0.545
Glycemic index (%)	87.0 ± 18.1	81.1 ± 15.8	0.075
Protein (g)	98.7 ± 16.0	97.0 ± 14.0	0.604
Total fat (g)	98.9 ± 16.3	105.8 ± 13.9	0.036
SFAs (g)	25.4 ± 5.1	26.8 ± 4.4	0.193
MUFAs (g)	50.2 ± 11.2	54.3 ± 9.9	0.050
PUFAs (g)	16.5 ± 4.1	17.8 ± 5.7	0.193
Omega 3 (g)	1.6 ± 0.4	1.7 ± 0.4	0.263
Omega 6 (g)	14.8 ± 4.1	16.0 ± 5.8	0.214
Trans fat (g)	1.0 ± 0.5	1.0 ± 0.5	0.864
Cholesterol (mg)	290.0 ± 75.4	287.7 ± 66.6	0.834
Palmitic acid (g)	15.7 ± 2.7	16.5 ± 2.2	0.090
Stearic acid (g)	5.9 ± 1.2	6.1 ± 1.1	0.545
Oleic acid (g)	47.7 ± 11.0	51.7 ± 9.7	0.050
Linoleic acid (g)	14.7 ± 4.1	15.9 ± 5.8	0.214
α-Linolenic acid (g)	1.1 ± 0.2	1.2 ± 0.2	0.214
Arachidonic acid (g)	0.2 ± 0.1	0.2 ± 0.0	0.604
EPA (g)	0.2 ± 0.1	0.2 ± 0.1	0.604
DHA (g)	0.3 ± 0.2	0.3 ± 0.2	0.604
Alcohol (g)	6.5 ± 11.6	5.6 ± 9.2	0.686
Caffeine (g)	244.0 ± 196.0	275.4 ± 233.9	0.601
Water (g)	2962.0 ± 720.0	2883.5 ± 676.8	0.604
Vitamin A (µg)	1283.0 ± 671.0	1212.1 ± 705.7	0.604
Retinol (µg)	355.0 ± 464.0	346.9 ± 374.3	0.887
Carotene (µg)	900.0 ± 436.0	841.6 ± 547.1	0.604
α Carotene (µg)	703.0 ± 535.0	610.2 ± 581.2	0.545
β Carotene (µg)	4868.0 ± 2 324.0	4578.6 ± 2 957.4	0.604
B Cryptoxanthin (µg)	332.0 ± 222.0	302.0 ± 190.4	0.602
Lutein+zeoxanthin (µg)	4147.0 ± 2 479.0	3951.2 ± 360.3	0.796
Lycopene (µg)	4457.0 ± 2 096.0	4292.6 ± 2 065.8	0.658
Folate (µg)	292.0 ± 73.0	283.7 ± 81.7	0.604
Vitamin B_12_ (mg)	8.3 ± 3.8	8.5 ± 3.5	0.796
Vitamin B_6_ (mg)	1.9 ± 0.5	1.9 ± 0.5	0.802
Vitamin C (mg)	116.0 ± 62.4	112.3 ± 55.6	0.796
Vitamin D (mg)	4.3 ± 1.7	4.4 ± 1.6	0.828
Vitamin E (mg)	13.9 ± 2.9	15.2 ± 3.6	0.050
Thiamine (mg)	1.6 ± 0.2	1.5 ± 0.3	0.075
Riboflavin (mg)	2.3 ± 0.5	2.2 ± 0.5	0.796
Niacin (mg)	27.9 ± 6.0	27.8 ± 6.3	0.860
Niacin equivalents (mg)	44.0 ± 8.0	43.6 ± 7.8	0.796
Calcium (mg)	1115.0 ± 329.0	1108.2 ± 317.7	0.887
Iron (mg)	13.1 ± 2.2	13.0 ± 2.8	0.796
Sodium (mg)	3381.0 ± 520.0	3449.8 ± 579.7	0.604
Potassium (mg)	3491.0 ± 695.0	3332.5 ± 652.4	0.262
Magnesium (mg)	415.0 ± 77.4	405.4 ± 84.4	0.604
Zinc (mg)	11.7 ± 1.7	11.4 ± 1.5	0.561
Selenium (µg)	145.0 ± 26.6	142.1 ± 24.0	0.604

^1^ Adjusted by energy intake. Data are means ± SD. ^2^ *p* was calculated by method of Benjamini and Hochberg. DR, diabetic retinopathy; SFAs, saturated fatty acids; MUFAs, monounsaturated fatty acids; PUFAs, polyunsaturated fatty acids; EPA, eicosapentaenoic acid; DHA, docosahexaenoic acid.

**Table 3:** Bivariate analysis for the relationship between the presence of diabetic retinopathy and clinical characteristics.

Characteristics	DR (*n* = 103)	No DR (*n* = 140)	OR [95% CI] ^1^	*p*
Sex (male)	48 (46.6)	62 (44.3)	1.10 [0.66;1.83]	0.722
Completed primary	36 (36.0)	34 (25.2)	1.75 [1.00; 3.09]	0.052
Secondary high school	39 (40.2)	49 (36.3)	1.17 [0.69; 1.99]	0.545
Graduate or higher	18 (18.6)	44 (32.6)	0.47 [0.25; 0.88]	0.018
Smoker, current	32 (31.1)	34 (24.3)	1.35 [0.76; 2.38]	0.241
Smoker, former	23 (22.3)	33 (23.6)	0.95 [0.52; 1.73]	0.820
Site Lleida	51 (49.5)	55 (39.3)	1.60 [0.96; 2.67]	0.113
Physical activity	77 (74.8)	98 (70.0)	1.27 [0.72; 2.27]	0.420
Age (years)	46.2 ± 10.8	42.1 ± 10.3	1.04 [1.01; 1.06]	0.004
BMI (kg/m^2^)	26.2 ± 4.3	25.3 ± 4.0	1.06 [0.99; 1.13]	0.073
Dyslipidemia	49 (47.6)	49 (35.0)	1.68 [1.00; 2.84]	0.050
Hypertension	40 (38.8)	20 (14.3)	3.77 [2.05; 7.13]	<0.001
Diabetes duration (years)	26.5 ± 9.9	17.9 ± 9.1	1.10 [1.07; 1.14]	<0.001
HbA1c (%)	7.9 ± 1.1	7.4 ± 0.8	1.97 [1.44; 2.69]	0.001

Data are mean ± SD or *n* (%). ^1^ Odds ratio and 95% confidence interval of diabetic retinopathy by clinical characteristics. DR, diabetic retinopathy; BMI, body mass index; HbA1c, glycated hemoglobin.

**Table 4:** Crude and adjusted multivariate analysis for the presence of diabetic retinopathy and daily nutrient intake.

Nutrients	DR (*n* = 103)	No DR (*n* = 140)	Crude OR [95% CI]	Crude *p*	Adjusted OR [95% CI] ^1^	Adjusted *p* ^1^
Carbohydrate (g)	198.0 ± 36.3	186.6 ± 31.2	1.01 [1.00; 1.02]	0.127	1.01 [0.99; 1.01]	0.127
Complex carbohydrate (g)	91.6 ± 18.6	85.8 ± 18.1	1.02 [1.00; 1.04]	0.031	1.02 [1.00; 1.04]	0.031
Glycemic index (%)	87.0 ± 18.1	81.1 ± 15.8	1.02 [1.00; 1.04]	0.087	1.02 [0.99; 1.04]	0.087
Total fat (g)	98.9 ± 16.3	105.8 ± 13.9	0.97 [0.94; 0.99]	0.009	0.96 [0.94; 0.98]	0.009
SFAs (g)	25.4 ± 5.1	26.8 ± 4.4	0.96 [0.90; 1.03]	0.290	0.97 [0.91; 1.03]	0.290
MUFAs (g)	50.2 ± 11.2	54.3 ± 9.9	0.96 [0.92; 0.99]	0.012	0.95 [0.92; 0.99]	0.012
Palmitic acid (g)	15.7 ± 2.7	16.5 ± 2.2	0.90 [0.78; 1.03]	0.126	0.89 [0.78; 1.02]	0.126
Oleic acid (g)	47.7 ± 11.0	51.7 ± 9.7	0.96 [0.92; 0.99]	0.012	0.95 [0.92; 0.99]	0.012
α-Linolenic acid (g)	1.1 ± 0.2	1.2 ± 0.2	0.16 [0.02; 1.23]	0.077	0.16 [0.02; 1.23]	0.077
Vitamin E (mg)	13.9 ± 2.9	15.2 ± 3.6	0.86 [0.77; 0.96]	0.006	0.85 [0.77; 0.95]	0.006
Thiamine (mg)	1.6 ± 0.2	1.5 ± 0.3	3.70 [0.86; 15.84]	0.078	3.70 [0.86; 15.84]	0.078

Crude and adjusted odds ratios and corresponding 95% confidence interval of diabetic retinopathy. Data are mean ± SD. ^1^ Adjusted by age, sex, educational level, smoking, center, physical activity, body mass index, dyslipidemia, hypertension, diabetes duration, and glycated hemoglobin. DR, diabetic retinopathy; SFAs, saturated fatty acids; MUFAs, monounsaturated fatty acids.

## Supplementary Materials

The following are available online at http://www.mdpi.com/2072-6643/10/9/1184/s1, Table S1: Daily food intake of participants according to diabetic retinopathy status.
