# Supplementary material for: Type 1 Diabetic Subjects with Diabetic Retinopathy Show an Unfavorable Pattern of Fat Intake
Source: Nutrients. 2018 Aug 29;10(9):1184. doi: 10.3390/nu10091184 (PMC6165451; doi:10.3390/nu10091184)
Supplement: Supplementary file 1 [file nutrients-10-01184-s001.pdf]

Table S1. Daily food intake of participants according to diabetic retinopathy status.

Daily Food Intake (g/day) [1]	DR (*n* = 103)	No DR (*n* = 140)	p [2]
Dairy products	415.7 ± 238.2	391.5 ± 245.8	0.742
Eggs	21.8 ± 14.8	19.8 ± 9.4	0.590
White meat	39.3 ± 23.9	34.4 ± 20.5	0.548
Red meat	53.3 ± 39.3	51.5 ± 38.9	0.935
Processed meat	47.6 ± 37.4	41.4 ± 27.4	0.548
Meat	137.0 ± 56.9	125.1 ± 53.5	0.548
Lean fish	32.8 ± 50.4	31.1 ± 23.7	0.935
Fatty fish	33.8 ± 25.9	39.0 ± 31.1	0.549
Seafood	9.2 ± 8.8	10.1 ± 8.6	0.742
Fish	76.3 ± 52.3	80.5 ± 46.3	0.789
Fruits and vegetables	493.0 ± 232.0	475.1 ± 236.7	0.789
Nuts	18.6 ± 37.3	12.1 ± 21.8	0.549
Legumes	34.0 ± 24.7	34.0 ± 24.6	0.983
Cereals and pasta	83.3 ± 41.1	82.3 ± 46.9	0.935
Potatoes	59.5 ± 39.7	53.1 ± 40.3	0.612
Bread	115.8 ± 57.3	96.6 ± 45.8	0.072
Sweets	15.5 ± 20.2	19.2 ± 21.6	0.549
Vegetable fats	37.0 ± 16.3	43.1 ± 16.6	0.072
Animal fats	0.2 ± 1.0	0.2 ± 1.1	0.935
Alcohol drinks	91.4 ± 165.0	93.9 ± 176.6	0.946
Non-alcoholic beverages	1 495.0 ± 612.0	1 549.9 ± 502.5	0.742
Coffee and tea	422.0 ± 262.0	428.0 ± 299.4	0.935
Prepared meals	77.6 ± 88.6	63.0 ± 81.0	0.549
Salt	1.0 ± 2.0	1.2 ± 1.7	0.732

Data are means ± SD. [1] Adjusted by energy intake. [2] P was calculated by method of Benjamini and Hochberg. DR, diabetic retinopathy.
